# Immunotherapy: A New Hope for Cancer Patients

**DOI:** 10.1155/2020/3548603

**Published:** 2020-07-09

**Authors:** Shalini Gupta, Subash C. Gupta, Keith D. Hunter, Aditya B. Pant

**Affiliations:** ^1^Department of Oral Pathology & Microbiology, King George's Medical University, Lucknow, India; ^2^Department of Biochemistry, Institute of Science, Banaras Hindu University, Varanasi, India; ^3^Academic Unit of Oral and Maxillofacial Medicine and Pathology, School of Clinical Dentistry, The University of Sheffield, Sheffield, UK; ^4^System Toxicology & Health Risk Assessment, CSIR-Indian Institute of Toxicology Research, Lucknow, India

Every year, a large number of people fall prey to cancer worldwide and more than half lose their battle to cancer. Among the multimodalities of cancer management, including surgery, hormonal therapy, radiotherapy, and chemotherapy, immunotherapy has revolutionized the treatment of cancer. Immune-oncology is a rapidly developing and exciting field of cancer treatment with the prospective of impacting the management of a wide array of malignancies which have recently witnessed a steep rise.

The major goal of cancer immunotherapy is to alleviate tumor-associated suppression of anticancer immune responses. The basic notion of utilizing a patient's immune system against cancer cells dates back to 1997, since the immune system response versus virulent cells during initial transformation in the immune surveillance process was discovered. Cancer immunotherapy, which is occasionally referred to as immune-oncology, induces the patient's own immune system and attempts to exploit the exquisite power and specificity of the immune system for cancer patient treatment. Immunotherapy against cancers encompasses a diverse continuum of approaches, ranging from stimulating effector mechanisms to counteracting inhibitory and suppressive mechanisms. Owing to the swift knowledge amassed by the scientific community about the immune system, small molecules, peptides, recombinant antibodies, vaccines, and cellular therapeutic modalities are being applied to manipulate the immune response to treat cancer.

With recent success, immunotherapy has emerged as a pragmatic strategy and a reputable pillar of cancer treatment improving the prognosis of many patients suffering from different malignancies. The two main drivers behind this success are immune checkpoint inhibitors (CPIs) and chimeric antigen receptor- (CAR-) T cells. For checkpoint blockade, current studies emphasize combinational approaches, perioperative use, response prediction, new tumor entities, toxicity management, and use in special patient populations. Regarding cellular immunotherapy, recent studies confirmed safety and efficacy of CAR-T cells in larger cohorts of patients with acute lymphoblastic leukemia or diffuse large B-cell lymphoma. Different strategies to translate the conspicuous triumph of CAR-T cells in B-cell malignancies to other hematological and solid cancer types are currently in clinical investigation phase. Since cancer is still one of the deadly challenges that are faced by humans, treatment regimes with single-drug therapies are not yielding very effective results in terms of improving the patient survival and treatment, and therefore, a combination of two or more therapies targeting different mechanisms could be more efficacious.

The purpose of this special issue is to present the recent progress in this exciting field. A brief summary of all accepted papers is provided as follows.

In the review article by M. Sambi et al., the authors have described the options to improve patient response rate to immunotherapy with an emphasis on adopting a multimodal approach with an emphasis on the novel role that the gut microbiota may play in modulating the efficacy of cancer immunotherapy.

The paper by D. D. Goycoechea et al. provides an extensive and critical review of the literature regarding immune checkpoint inhibition in classical Hodgkin lymphoma. The paper discusses the early achievements and provides new perspectives on immune checkpoint inhibition in a disease that constitutes a model of sensitivity to this treatment.

The paper by A. Rogiers et al. presents an updated and comprehensive review of the results of the main clinical trials in the field of immunotherapy in melanoma and highlights potential consequences on the psychosocial wellbeing, neurocognitive functions, and life quality of patients.

In the research article by P. Darvin et al., the authors investigated the regulation of PD-L1 expression in breast cancer tumor spheres compared to cell lines. They show that PD-L1 is likely regulated by histone modifications rather than DNA methylation.

In the paper by D. Wu et al., the authors reached the conclusion that CTP is currently the most effective neoadjuvant regimen for the chance of achieving pathological complete response (pCR), with little additional toxicity compared, and MP has the best tolerability and acceptable efficacy.

In the research article by A. Rossi et al., the authors point out that ALDH levels are elevated in the serum of NSCLC patients with advanced stage as well as in early stage disease and therefore can be evaluated as part of the panel of markers for noninvasive detection of early NSCLC in a larger cohort of patients at risk.

In the paper by A. C. Martínez-Torres et al., the authors have discovered that PKHB1, a TSP1-derived CD47 agonist peptide, can induce immunogenic cell death in tumoral T lymphoblasts. Tumor cell lysate (TCL) from PKHB1-treated lymphoblasts can induce DC maturation and T-cell activation ex vivo. Also, TCL can be used as tumor vaccine, when administrated in either prophylactic or therapeutic settings.

In the review article by C. Fiorentini et al., the authors have reviewed clinical results of immunotherapy in ACC and highlighted molecular mechanisms that lead to immunotherapy failure in ACC. They have also suggested possible approaches to prevail over resistance.

In the review article by R. E. Cooke et al., the authors have discussed the concept of immune profiling to target patients of multiple myeloma who might benefit the most from immunotherapeutics. The review also covers the advances and use of humanized mice as well as 3D culture systems for personalized medicine.

In the paper by M. Kreidieh et al., the authors have reviewed in detail about treatment strategy in liver cancer including radiation therapy and immunotherapy alone and in combination. The study also explores the evidence surrounding the use of SBRT and immunotherapy for the treatment of HCC and CCA.

In the paper by F. Toia et al., the authors have presented a systematic review of preclinical and clinical studies on *γδ* T cell-based immunotherapy and melanoma, in which the discussion is mainly focused on research state of the art and future perspectives.

In the review article by F. Ghali et al., the authors have reviewed the current status of adjuvant and neoadjuvant immunotherapy in localized and locally advanced renal cell carcinoma, combining discussion of recently published, ongoing clinical trials and future concepts in this fast moving area of investigation.

In the paper by M. A. I. Al-Hatamleh et al., the authors have reviewed the implications of tumour necrosis factor and its receptor 2 (TNFR2) expressions in breast cancer, the oncogenic consequences, and their role in the suppressive immune responses by TRegs. They have described the use of nanoparticles as a targeted drug delivery agent in immunotherapy, based on their advantageous properties. The review also discusses the manipulation of NPs with TNF-antagonist to modulate TNF-TNFR2 interaction that inhibits breast cancer progression.

In the research article by E.-G. Chavez-Cortez et al., the authors show a novel, affordable, and effective tumor, glioblastoma. The immunotoxin produced and tested in this investigation, i.e., IgY-abrin immunotoxin, had cytotoxic activity against CD133+ malignant glioma stem cells and provided a novel approach for the immunotherapy of glioblastoma.

The research article by U. Swami et al. suggests that advanced melanoma patients discontinuing anti-PD-1 therapy due to irAE usually experience durable clinical benefit. However, caution is needed with these agents in patients with underlying autoimmune diseases. The study explored durable clinical benefit in this subset of patients. The manuscript goes in detail of each immune-toxicity and its management.

The review by S. P. Singh et al. provides an interesting overview of the role of cancer stem cells and molecular pathways that are aberrantly expressed in these cells. This review article also provides an updated description of markers expressed by cancer stem cells and used for their isolation. It also covers the options of treatment of cancer stem cells with immunotherapy. Therefore, examples of molecules/pathways expressed by cancer stem cells that could represent target for immunotherapy approaches are also included in the review.

